# Anxiety- and depressive-like responses and c-*fos *activity in preproenkephalin knockout mice: Oversensitivity hypothesis of enkephalin deficit-induced posttraumatic stress disorder

**DOI:** 10.1186/1423-0127-17-29

**Published:** 2010-04-21

**Authors:** Jen-Chuang Kung, Tsung-Chieh Chen, Bai-Chuang Shyu, Sigmund Hsiao, Andrew Chih Wei Huang

**Affiliations:** 1 Institute of Biomedical Sciences, Academia Sinica, Taipei 11529, Taiwan, Republic of China; 2 Department of Psychology, National Chung Cheng University, Chia-Yi 168, Taiwan, Republic of China; 3 Department of Medicine, Kaohsiung Medical University, Kaohsiung, Taiwan, Republic of China; 4 Department of Psychology, Fo Guang University, Yi-Lan 26247, Taiwan, Republic of China

## Abstract

The present study used the preproenkephalin knockout (ppENK) mice to test whether the endogenous enkephalins deficit could facilitate the anxiety- and depressive-like symptoms of posttraumatic stress disorder (PTSD). On Day 1, sixteen wildtype (WT) and sixteen ppENK male mice were given a 3 mA or no footshock treatment for 10 seconds in the footshock apparatus, respectively. On Days 2, 7, and 13, all mice were given situational reminders for 1 min per trial, and the freezing response was assessed. On Day 14, all mice were tested in the open field test, elevated plus maze, light/dark avoidance test, and forced swim test. Two hours after the last test, brain tissues were stained to examine c-*fos *expression in specific brain areas. The present results showed that the conditioned freezing response was significant for different genotypes (ppENK vs WT). The conditioned freezing effect of the ppENK mice was stronger than those of the WT mice. On Day 14, the ppENK mice showed more anxiety- and depressive-like responses than WT mice. The magnitude of Fos immunolabeling was also significantly greater in the primary motor cortex, bed nucleus of the stria terminalis-lateral division, bed nucleus of the stria terminalis-supracapsular division, paraventricular hypothalamic nucleus-lateral magnocellular part, central nucleus of the amygdala, and basolateral nucleus of the amygdala in ppENK mice compared with WT mice. In summary, animals with an endogenous deficit in enkephalins might be more sensitive to PTSD-like aversive stimuli and elicit stronger anxiety and depressive PTSD symptoms, suggesting an oversensitivity hypothesis of enkephalin deficit-induced PTSD.

## Background

Posttraumatic stress disorder (PTSD) shows a variety of symptoms including the exaggerated fear, helplessness, and horror after patients suffer from an extremely stressful traumatic event (an unconditioned stimulus [US]) [1]. For example, the reexperiencing of symptoms of an earlier traumatic event includes panic attack, phobic avoidance of situations that resemble the traumatic event, and psychic numbing [2,3]. Additional symptoms comprise autonomic hyperarousal responses and fear sensitization, such as exaggerated startle responses, hypervigilance, insomnia, irritability, and impaired concentration [4].

In addition to the traumatic event US inducing PTSD-like responses, the environmental stimulus (conditioned stimulus [CS]) associated with the traumatic event US is also able to elicit PTSD-like avoidance fear responses [5]. Accordingly, an animal model of PTSD has been developed in which individuals are repeatedly exposed to situational reminders that have been previously associated with a traumatic stress US to elicit the fear response [6,7].

The PTSD-like symptoms have been shown to be governed by specific neurotransmitters [8,9]. For example, a recent report has demonstrated that the releasing concentrations of serotonin, norepinephrine, and dopamine in the hippocampus and frontal cortex would be enhanced, and the plasma corticosterone levels in the hypothalamic-pituitary-adrenal axis were increased after acute stress exposure [10]. Moreover, a recent study demonstrated overactivity of norepinephrine and vasopressin systems, and deficits of glucocorticoid and serotonin systems resulted in a cognitive syndrome resembling PTSD [11].

Additionally, several lines of evidence suggest that the opioid system is also involved in PTSD. When reencountering a traumatic stressor, PTSD patients exhibit an increased endogenous opioid-mediated and stress-induced analgesic effect [7,12,13]. The pain mechanism is also associated with PTSD-like symptoms, particularly associative fear conditioning [14,15]. Further evidence is provided by ppENK knockout mice. These mice are deficient in enkephalin, an opioid peptide, and are prone to heightened anxiety-like behavior, stress reactions, and aggressive responses [16] compared with WT mice. In contrast, the mice in over-expression with ppENK in the amygdala could induce the anxiolytic effect [17]. Additionally, enkephalins have been shown to be associated with postsynaptic μ- and δ-opioid receptors to affect supraspinal and spinal analgesia [18]. Thus, μ- and δ-opioid receptors are probably to be involved in stress-induced PTSD-like symptoms [18,19]. These results suggest that enkephalins may be involved in PTSD-like symptoms.

The present study examined whether endogenous enkephalins play a crucial role in PTSD. ppENK mice were compared with WT mice in a PTSD-like footshock trauma recall paradigm. This animal model of PTSD was designed to expose animals to a traumatic footshock stimulus in a specific context on Day 1 and to later reexpose the animals to the same context without footshock on Days 2, 7, and 13. Conditioned freezing behavior was then measured. During the test session (Day 14), all mice were tested for numerous anxiety-like responses in the elevated plus maze, light/dark avoidance test, and open field test. PTSD is often comorbid with depressive disorders, and depressive behaviors were also measured in these animals in the forced swim test [20-22]. Two hours later, Fos immunohistochemistry was performed to examine which brain nuclei may be involved in PTSD-like symptoms.

## Methods

### Animals

Sixteen WT C57BL/6J male mice were obtained from the Experimental Animal Center for Academia Sinica, Taipei, Taiwan. Sixteen ppENK male mice (B6.129-*Penk-rs*^*tm1Pig*^; background strain C57BL/6J) were purchased from Jackson Laboratories (Bar Harbor, ME, USA). The primer sets used to identify both WT and ppENK alleles have been previously described [16,23].

All mice weighed 25-35 g at the beginning of the experiment. Mice were group-housed, five per cage, in a colony room with a controlled 12:12 hr light/dark cycle, with lights on from 0600-1800 h. The colony room was maintained at 22°C, and mice were given *ad libitum *access to food and water. All experiments were performed in compliance with the Animal Scientific Procedures Act of 1986 and received local ethics committee approval. Efforts were made to minimize animal suffering and the number of animals used.

### Behavioral procedure

**Figure 1:**
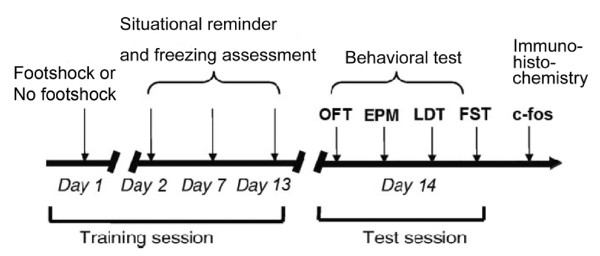
**Diagram showing the experimental design**. On Day 1, seven WT and seven ppENK mice received footshocks (3 mA) for 10 s to induce a traumatic event. Nine WT and nine ppENK mice received no footshocks. On Days 2, 7, and 13, all mice were exposed to situational reminders, and freezing behavior was recorded. On Day 14, each mouse underwent the following behavioral tests: open field test, elevated plus maze, light/dark avoidance test, and forced swim test. Two hours later, mice were euthanized and examined for Fos immunolabeling in multiple brain areas.

On Day 1, seven WT and seven ppENK mice were given a single footshock in the footshock apparatus; another nine WT and nine ppENK mice did not receive footshocks. On Days 2, 7, and 13, all mice were exposed to situational reminders, which consisted of placement in the footshock apparatus without any footshock. During the situational reminder treatment, freezing behavior was recorded. On Day 14, all mice were given the following four behavioral tests: open field test, elevated plus maze, light/dark test, and forced swim test. The order of behavioral testing was random. Two hours after the last test, mice were euthanized, and their brains were processed for Fos immunolabeling [24] (Fig. 1).

### Apparatus and induction of associative fear

#### Inescapable footshock

The inescapable footshock apparatus was a box composed of a plastic surrounding shell measuring 29 cm × 29 cm × 36 cm high. The floor of the apparatus was composed of metal grids (0.3 cm diameter at 0.7 cm grid intervals). On Day 1, sixteen WT and sixteen ppENK mice were exposed to this apparatus for 2 min. Seven WT and seven ppENK were then given a 3 mA footshock (duration, 10 second), and another nine WT and nine ppENK mice received no footshock [25]. The single strong footshock treatment was referenced and modified by previous reports [6,26-28].

#### Situational reminders

Situational reminders were given on Days 2, 7, and 13. During these sessions, all mice reencountered the footshock environment without footshock for 1 min once per day. Such a condition was designed to mimic the continuous and repeated suffering of traumatic events experienced by human PTSD patients [29].

### Behavioral testing

#### Elevated plus maze

The apparatus included two open arms (30 cm long × 5 cm wide) and two closed arms (30 cm long × 5 cm wide × 15 cm high). The open and closed arms were made of dark plastic material and were perpendicular. The halfway point of the intersection was 5 cm^2^, and the apparatus was raised 50 cm from the floor with four plastic sticks. These measures of anxious behavior in the elevated plus maze task were almost followed by the method of Melchior and Ritzmann (1994). At the beginning of each testing, the mouse was put at one end of one of the open arms. A mouse's latency time to reach the halfway point was recorded. Larger latency time indicated the greater avoidance and the more strength of anxiety. Also, the number of entries into the open arms was measured for 3 min. Smaller scores of entries into the open arm indicated the more strength of anxiety. An entry was defined as placing at least two paws into the open arm [30].

#### Light/dark avoidance test

The apparatus was composed of a set of light and dark plastic chambers (17 × 16 × 15 cm high for each chamber) separated by a partition (11.5 cm long × 0.3 cm wide × 13 cm high). The dark chamber was designed similarly to the inescapable footshock environment, which had electric grids (16 cm long × 0.3 cm diameter × 0.7 cm grid intervals) on the ground, to generalize between the two environments. The light chamber included a 60 watt white light and wire nets on the ground. The latency time was recorded for 5 min. When mice did not enter the dark chamber for 5 min, the latency time was recorded as 5 min. Larger scores of latency time indicated the stronger anxiety behavior [31].

#### Open field test

The apparatus consisted of a square arena (80 cm long × 80 cm wide × 40 cm high) with a 40 cm^2 ^inner area. When a mouse was placed in one corner of the outer area, it was allowed to explore the arena for 10 min. The time spent in the inner area and entries into the inner area were recorded. Less time spent in the inner area or fewer entries into the inner area indicated the stronger anxiety behavior [32].

#### Forced swim test

The forced swim apparatus consisted of a glass cylinder (18 cm diameter, 27 cm high) filled with warm water (about 25°C) to a depth of 15 cm. The forced swim test was designed such that each mouse could not float with the hind legs touching the bottom. For each trial, subjects were gently placed into the water for 5 min and then the subjects were returned to their home cage. The duration of floating (defined as an absence of movement with the exception of movements necessary to keep the head above the water), swimming (defined as forward motion through the water and forepaws kept at the water surface), and struggling (defined as an upright position in the water and forepaws breaking the water surface) were scored. Larger scores of floating, smaller scores of swimming, and smaller scores of struggling indicated the stronger depressive behavior [33].

#### Freezing behavior

Freezing behavior indicated the fear response and was defined as the absence of movement with the exception of respiration. Also, greater scores of freezing behavior represented the greater strength of the fear response [34]. In the present study, the freezing behavior occurred when an animal was exposed to an environmental stimulus (i.e. CS) that had been paired with traumatic stimuli (i.e. US). When rats encountered the previous CS alone, the so-called situational reminder procedure could elicit a conditioned fear response. A video camera recorded conditioned fear responses during exposure to the situational reminders on Days 2, 7, and 13.

#### c-*fos *expression

The expression of c-*fos*, an immediate early gene reflecting neural activity, was assessed by measuring Fos immunoreactivity [24]. Two hours after assessment of anxiety-like and depressive-like behavior (on Day 14), mice were euthanized with an overdose of pentobarbital injected intraperitoneally. Mice were then transcardially perfused with 150 ml of 0.9% saline followed by 150 ml of 4% paraformaldehyde in 0.1 M phosphate-buffered saline (PBS, pH 7.4). After perfusion, the brain was removed and postfixed overnight in 4% paraformaldehyde at 4°C. The brain was then immersed in a 30% sucrose solution for 48 h.

Brains were then frozen and sliced in 50 μm coronal sections on a freezing microtome maintained at -20°C. Brain sections were collected and immersed in a 0.1 M PBS solution. Anterior and posterior orientation was guided by the Paxinos and Franklin mouse brain atlas [35].

Sections were then processed for Fos-LI. Sections were first incubated in an antigen retrieval solution (0.1 M PBS, 100% methanol, and 3% H_2_O_2_) for 30 min. Sections were then washed in 0.1 M PBS for 3 × 10 min and incubated in 3% normal goat serum containing 0.1% triton (NGST) for 1 h to block nonspecific antigens. Sections were then transferred to a primary antibody solution of rabbit anti-Fos antibody in 1% NGST (1:1000, Santa Cruz) and incubated at 4°C for 24 h. After rinsing in 0.1 M PBS for 10 min, sections were incubated in secondary antibody, a solution of goat biotinylated anti-rabbit IgG in 1% NGST (1:200, Vector, BA-1000) for 1 h. After again rinsing in 0.1 M PBS for 10 min, sections were incubated in an avidin-biotin elite solution in PBS (ABC kit, Vector, CA) for 1 h. Another rinse in 0.1 M PBS was then followed by incubation in a chromogen reaction solution (Tris, pH 7.4, 3% H_2_O_2_, and 0.03% 3,3'-diaminobenzidine) for 10 min. Finally, all sections were rinsed in PBS solution and mounted onto gelatin-coated slides.

### Data quantification and analysis

#### c-*fos *expression

Quantitative analysis of Fos-LI was performed on sections selected by a technician blind to experimental treatments. For each brain, consecutive sections showing positive dark brown immunoreactivity at 20× magnification were chosen by two observers blind to the experimental treatment. In each section, the number of cells with Fos-LI was counted bilaterally in the candidate brain areas (which were likely involved in PTSD-like symptoms) of tissue measuring 200 μm^2^. Average cell counts were calculated for each subject. Sixteen candidate brain areas were analyzed, but only six brain areas showed significant differences between WT and ppENK mice (Table 1). Cell count data were tested for significant differences between the factors of genotype and footshock by two-way ANOVA, depending on the specific brain areas. Values of *p *< 0.05 were considered statistically significant. All data are expressed as mean ± standard error.

**Table 1:** Analysis of Genotype and Footshock factors and interactions in ppENK and WT mice in the following brain areas using 2 × 2 two-way ANOVA: VO, M1, PrL & IL, BSTL, AC, PVT, BNST, PaLM, LH, Cg/RS, CeA, BLA, MeA, DG, CA1, and CA2.

Factors Brain areas	Subjects (Wild type vs ppENK)	Footshocks (No footshocks vs Footshock)	An interaction of subjects and footshocks
**VO**	F(1,28) = 2.86, p > 0.05	F(1,28) = 0.14, p > 0.05	F(1,28) = 0.02, p > 0.05

**M1**	F(1,28) = 9.00, p > 0.05*	F(1,28) = 5.46, p > 0.05*	F(1,28) = 0.56, p > 0.05

**Prl & IL**	F(1,28) = 3.14, p > 0.05	F(1,28) = 0.41, p > 0.05	F(1,28) = 1.86, p > 0.05

**BSTL**	F(1,28) = 7.55, p > 0.05*	F(1,28) = 1.12, p > 0.05	F(1,28) = 0.69, p > 0.05

**AC**	F(1,28) = 0.65, p > 0.05	F(1,28) = 0.04, p > 0.05	F(1,28) = 0.04, p > 0.05

**PVT**	F(1,28) = 0.03, p > 0.05	F(1,28) = 1.99, p > 0.05	F(1,28) = 0.13, p > 0.05

**BNST**	F(1,28) = 12.19, p > 0.05*	F(1,28) = 6.33, p > 0.05*	F(1,28) = 0.18, p > 0.05

**PaLM**	F(1,28) = 3.54, p > 0.05	F(1,28) = 0.12, p > 0.05	F(1,28) = 6.56, p > 0.05*

**LH**	F(1,28) = 0.00, p > 0.05	F(1,28) = 9.00, p > 0.05*	F(1,28) = 2.25, p > 0.05

**Cg/RS**	F(1,28) = 3.27, p > 0.05	F(1,28) = 3.16, p > 0.05	F(1,28) = 1.34, p > 0.05

**CeA**	F(1,28) = 4.67, p > 0.05*	F(1,28) = 5.49, p > 0.05*	F(1,28) = 0.28, p > 0.05

**BLA**	F(1,28) = 6.61, p > 0.05*	F(1,28) = 5.61, p > 0.05*	F(1,28) = 0.55, p > 0.05

**MeA**	F(1,28) = 1.98, p > 0.05	F(1,28) = 0.87, p > 0.05	F(1,28) = 3.12, p > 0.05

**DG**	F(1,28) = 0.99, p > 0.05	F(1,28) = 0.99, p > 0.05	F(1,28) = 0.13, p > 0.05

**CA1**	F(1,28) = 0.00, p > 0.05	F(1,28) = 0.10, p > 0.05	F(1,28) = 0.37, p > 0.05

**CA2**	F(1,28) = 1.61, p > 0.05	F(1,28) = 0.14, p > 0.05	F(1,28) = 0.00, p > 0.05

*p < 0.05, significant difference.

### Behavioral data analysis

Data obtained from the four behavioral tests (elevated plus maze, light/dark test, open field test, and forced swim test) were analyzed by a 2 × 2 two-way ANOVA with the factors of genotype and footshock. Conditioned freezing behavior was analyzed by a mixed 2 × 2 × 3 three-way ANOVA with repeated sessions. When appropriate, *post hoc *tests were conducted using Tukey's Honestly Significant Difference test. A *p *value less than 0.05 was considered significant and labeled with one star (*). A *p *value higher than 0.05 was seen to be not significant and labeled with marks (ns). All data are expressed as mean ± standard error.

## Results

### Freezing behavior during situational reminders

**Figure 2:**
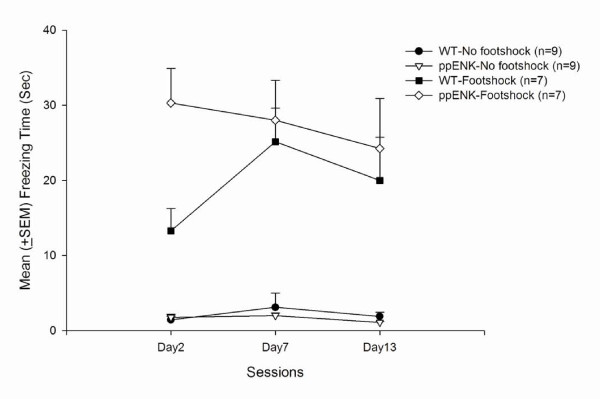
**Mean (± SEM) freezing time during situational reminders on Days 2, 7, and 13**. Conditioned freezing behavior was assessed in WT-no footshock, ppENK-no footshock, WT-footshock, and ppENK-footshock groups.

The magnitude of conditioned freezing behavior was measured after only one trial of footshock or no footshock treatments (Fig. 1). A 2 × 2 × 3 mixed three-way repeated-measures analysis of variance (ANOVA) (factors Genotype, Footshock, and Session) indicated Footshock was significant (*F*_1,28 _= 102.86, *p *< 0.05). Moreover, Genotype × Footshock interaction was significant (*F*_1,28 _= 4.08, *p *= 0.05). However, the p value of Genotype was approximately near to 0.05 (*F*_1,28 _= 3.15, *p *= 0.09). Sessions did not have a significant effect (*F*_2,56 _= 1.19, *p *> 0.05). Additionally, Session × Genotype interaction was not significant (*F*_2,56 _= 2.07, *p *> 0.05). Session × Footshock interaction was not significant (*F*_2,56 _= 0.05, *p *> 0.05). Genotype × Footshock × Session interaction was not significant between all three variables (*F*_2,56 _= 1.40, *p *> 0.05). Thus, we suggest that different genotypes (ppENK vs WT) probably had different freezing responses. Furthermore, ppENK mice were probably stronger freezing effect relative to the WT mice. Footshock treatments actually produced conditioned freezing responses. Moreover, there were significant interactions between Genotype and Footshock (Fig. 2). The present results mean that different genotypes have different conditioned freezing responses underlying an appropriate footshock treatment. The conditioned freezing behavior of the ppENK mice showed stronger than those of the WT mice.

### Anxiety measure: elevated plus maze

**Figure 3:**
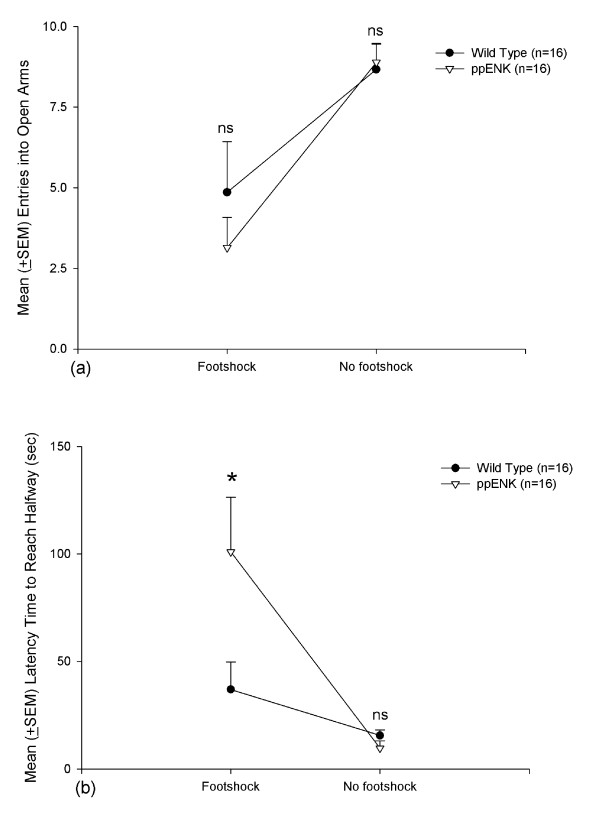
**Elevated plus maze**. (a) Mean (± SEM) entries into open arms and (b) mean (± SEM) latency time to reach halfway in WT-footshock (*n *= 7), ppENK-footshock (*n *= 7), WT-no footshock (*n *= 9), and ppENK-no footshock (*n *= 9) groups. * *p *< 0.05 and n.s. are significant and non-significant (*p *> 0.05) when comparing the significant difference between wild type and ppENK groups.

The mean (± SEM) entries into the open arms from closed arms and mean (± SEM) time spent halfway from the open arms indicated anxiety-like responses in WT and ppENK mice in the elevated plus maze test. The ppENK groups did not exhibit significant differences in entries from the closed to open arms during the 3 min test compared with the WT groups (*F*_1,28 _= 0.59, *p *> 0.05). However, footshock treatment elicited a significant difference (*F*_1,28 _= 24.13, *p *< 0.05). No Genotype × Footshock interaction was found (*F*_1,28 _= 0.99, *p *> 0.05). Furthermore, no significant effects were observed between WT-footshock and ppENK-footshock groups or between WT-no footshock and ppENK-no footshock groups (*p *> 0.05) (Fig. 3a). However, the time spent halfway from the open arms was significantly greater in ppENK mice compared with WT mice (*F*_1,28 _= 5.25, *p *< 0.05), with a significant effect of footshock treatment between the WT and ppENK groups (*F*_1,28 _= 19.78, *p *< 0.05). A significant Genotype × Footshock interaction was also found (*F*_1,28 _= 7.60, *p *< 0.05). Moreover, *post hoc *comparisons indicated that ppENK-footshock mice required more time to reach halfway to the open arms compared with WT-footshock mice (*p *< 0.05) (Fig. 3b). Thus, the index of time spent to reach the halfway point in the elevated plus maze test was seemingly more sensitive than the index of entries into the open arms, particularly in ppENK mice, when assessing anxiety-like responses. Overall, ppENK mice exhibited more anxiety-like behavior than WT mice in the elevated plus maze test.

### Anxiety measure: light/dark avoidance test

**Figure 4:**
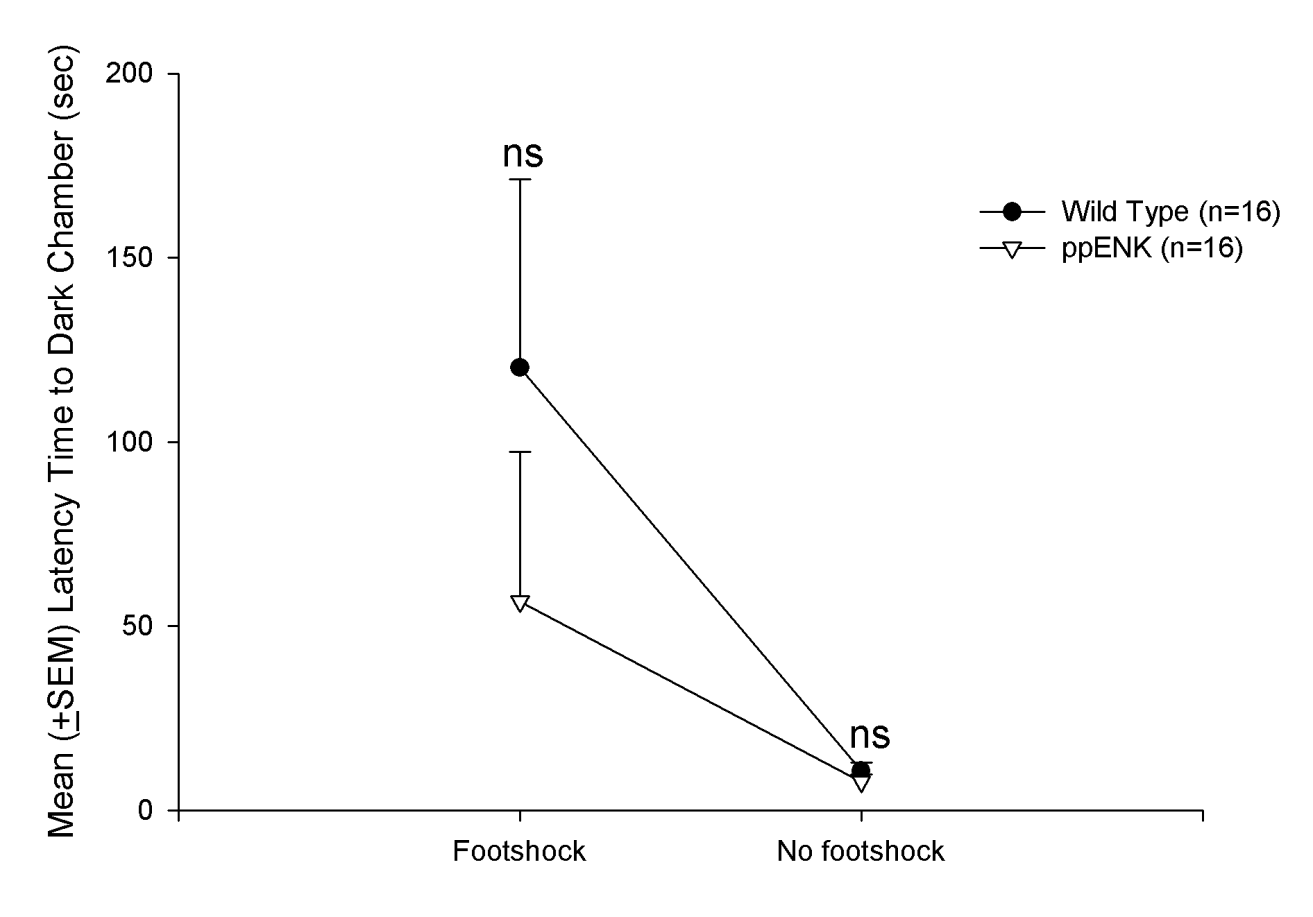
**Light/dark avoidance test**. Mean (± SEM) latency time prior to entering the dark chamber in WT-footshock (*n *= 7), ppENK-footshock (*n *= 7), WT-no footshock (*n *= 9), and ppENK-no footshock (*n *= 9) groups. n.s. indicates there is a non-significant (*p *> 0.05) when comparing the significant difference between wild type and ppENK groups.

The mean (± SEM) latency to enter the dark chamber was measured during a 5 min period. WT and ppENK mice did not exhibit significant differences in latency (*F*_1,28 _= 1.35, *p *> 0.05). A significant effect of Footshock was observed (*F*_1,28 _= 7.67, *p *< 0.05), with a non-significant Genotype × Footshock interaction (*F*_1,28 _= 1.12, *p *> 0.05). Thus, the different genotype mice (ppENK v.s. WT mice) did not affect latency time to enter the dark compartment, regardless of the footshock and no footshock conditions (Fig. 4).

### Anxiety measure: open field test

**Figure 5:**
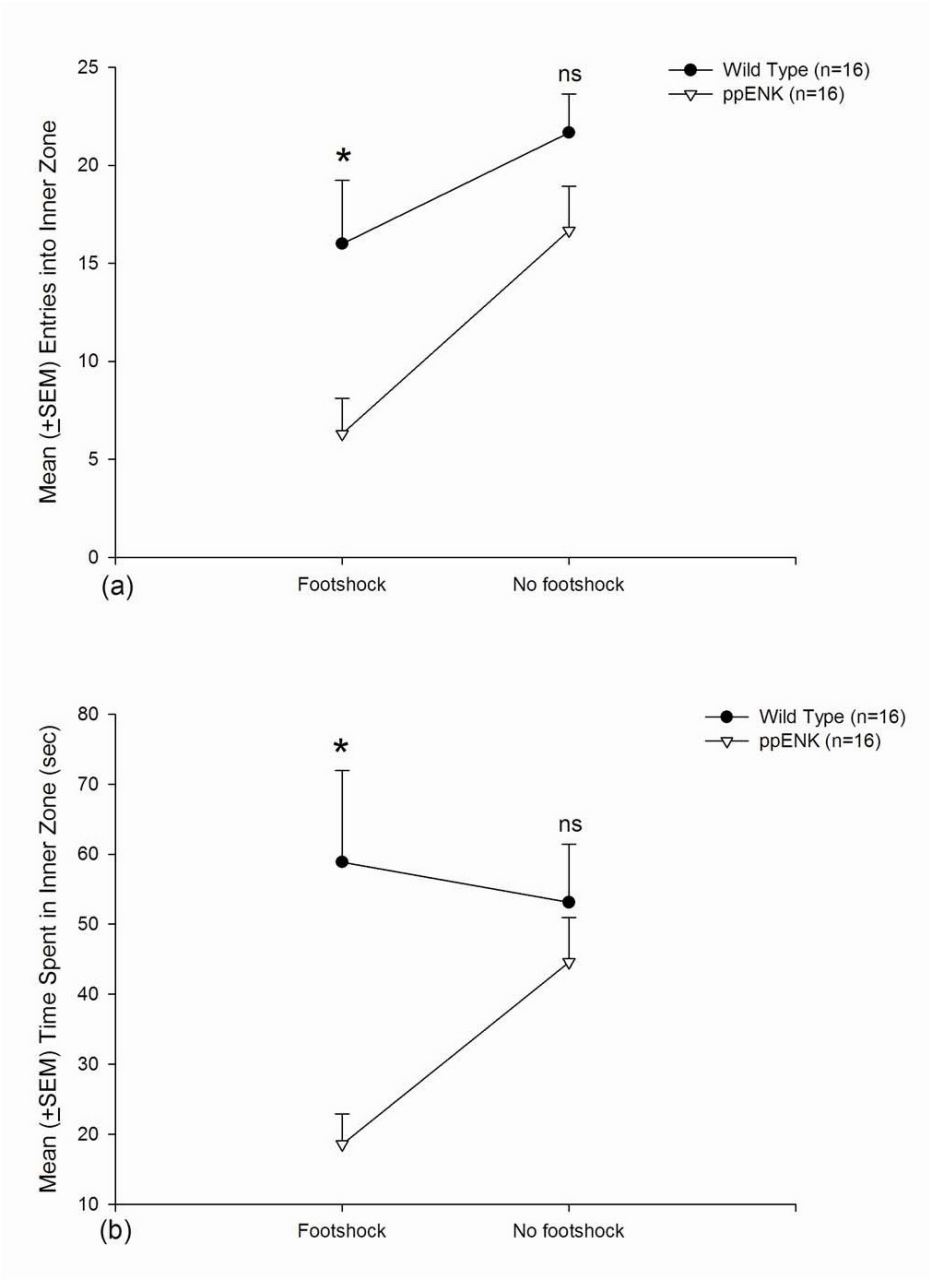
**Open field test**. (a) Mean (± SEM) entries into the inner area and (b) mean (± SEM) time spent in the inner area in WT-footshock (*n *= 7), ppENK-footshock (*n *= 7), WT-no footshock (*n *= 9), and ppENK-no footshock (*n *= 9) groups. * *p *< 0.05 and n.s. are significant and non-significant (*p *> 0.05) when comparing the significant difference between wild type and ppENK groups.

During the 10 min test, two anxiety-like responses were tested: entries into the inner area and time spent in the inner area. ppENK mice exhibited significantly fewer entries into the inner area compared with WT mice (*F*_1,28 _= 9.71, *p *< 0.05), with a significant effect of Footshock (*F*_1,28 _= 11.55, *p *< 0.05) but no Genotype × Footshock interaction (*F*_1,28 _= 1.00, *p *> 0.05). *Post hoc *comparisons indicated that ppENK-footshock mice made fewer entries into the inner area compared with WT-footshock mice (*p *< 0.05). However, this effect was not observed when comparing ppENK-no footshock and WT-no footshock mice (Fig. 5a). ppENK mice spent significantly less time in the inner area compared with WT mice (*F*_1,28 _= 8.27, *p *< 0.05), and this measure was not significantly affected by footshock (*F*_1,28 _= 1.42, *p *> 0.05). No Genotype × Footshock interaction was found (*F*_1,28 _= 3.49, *p *> 0.05). *Post hoc *comparisons indicated that ppENK-footshock mice spent significantly less time in the inner area compared with WT-footshock mice (*p *< 0.05), but this effect did not occur in the no footshock condition (Fig. 5b). The entries into and time spent in the inner area in ppENK mice were significantly decreased compared with WT mice, especially in the footshock condition. Thus, the entries into and time spent in the inner area of the open field test were both valid indices for assessing anxiety-like behavior.

### Depressive measure: forced swim test

**Figure 6:**
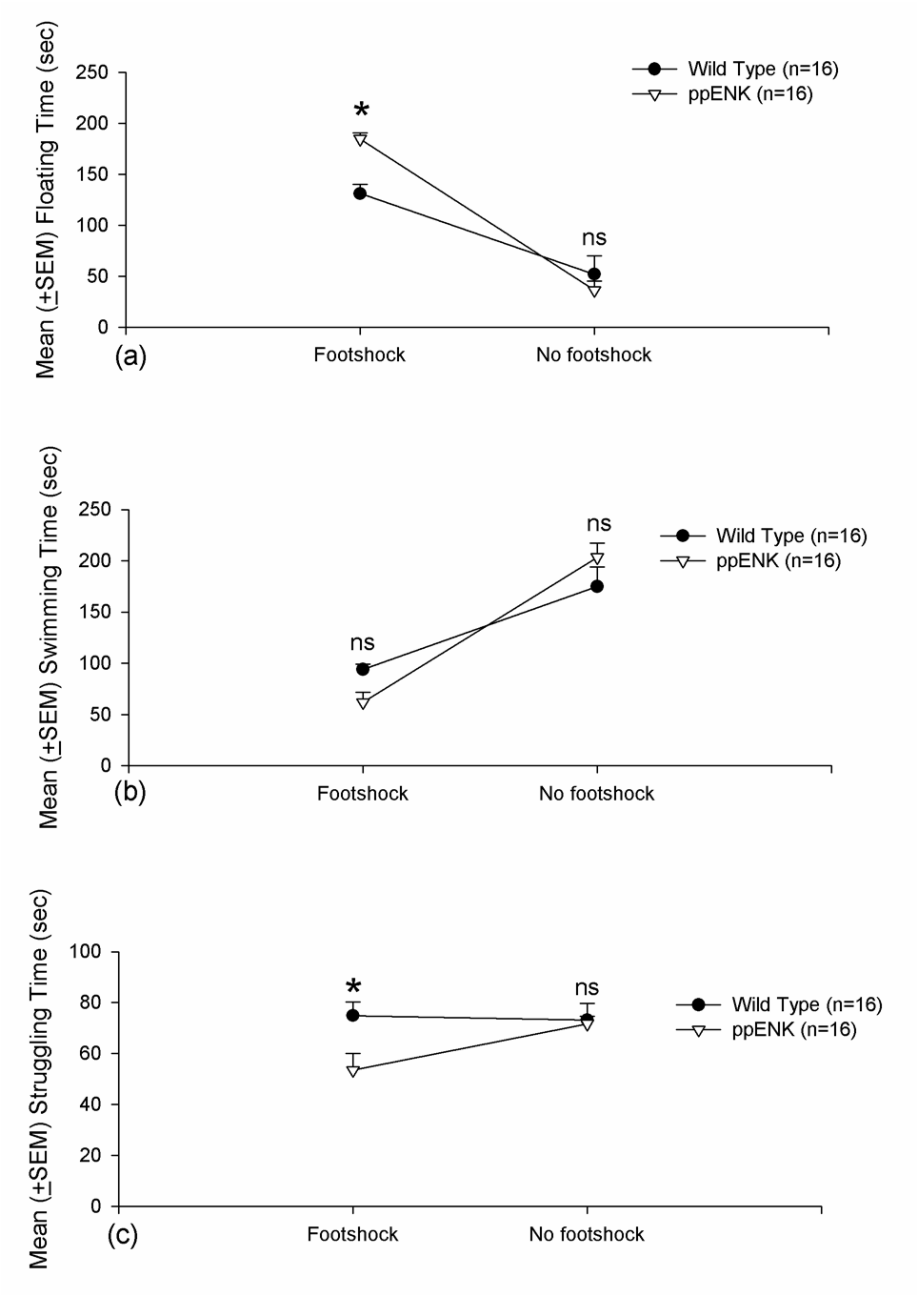
**Forced swim test**. (a) Mean (± SEM) floating time, (b) mean (± SEM) swimming time, and (c) mean (± SEM) struggling time in WT-footshock (*n *= 7), ppENK-footshock (*n *= 7), WT-no footshock (*n *= 9), and ppENK-no footshock (*n *= 9) groups. * *p *< 0.05 and n.s. are significant and non-significant (*p *> 0.05) when comparing the significant difference between wild type and ppENK groups.

Learned helplessness behavior, such as floating, swimming, and struggling, were measured during a 5 min period, with the factors Genotype and Footshock. No significant difference in floating behavior was observed between WT and ppENK mice (*F*_1,28 _= 2.31, *p *> 0.05). However, a significant effect of Footshock was observed (*F*_1,28 _= 83.98, *p *< 0.05), with a significant Genotype × Footshock interaction (*F*_1,28 _= 7.78, *p *< 0.05). *Post hoc *comparisons indicated that ppENK-footshock mice had increased floating time compared with WT-footshock mice (*p *< 0.05) (Fig. 6a). Swimming time was also not significantly different between WT and ppENK mice (*F*_1,28 _= 0.02, *p *> 0.05). Significant effect of Footshock was observed between WT and ppENK mice (*F*_1,28 _= 60.21, *p *< 0.05), but a significant Genotype × Footshock interaction was observed (*F*_1,28 _= 4.47, *p *< 0.05). *Post hoc *comparisons indicated no significant effect between ppENK-footshock and WT-footshock groups (*p *> 0.05) (Fig. 6b). Significantly less struggling time was observed in ppENK mice compared with WT mice (*F*_1,28 _= 4.35, *p *< 0.05). No significant effect of Footshock was observed on struggling behavior (*F*_1,28 _= 2.27, *p *> 0.05), with no significant Genotype × Footshock interaction (*F*_1,28 _= 3.25, *p *> 0.05). *Post hoc *comparisons indicated a trend toward a significant difference between ppENK-footshock and WT-footshock mice (*p *= 0.07), but this difference did not occur in the no footshock condition (Fig. 6c). Thus, only struggling time was determined to be a better depressive index for dissociating WT and ppENK mice. In contrast, floating time and swimming time were not sufficient for discriminating depressive-like behavior between WT and ppENK mice.

### c-*fos *analysis

Fos immunolabeling revealed the activation of brain areas after testing the four anxiety and depressive tasks in the present study. Because a 2 × 2 two-way ANOVA with the factors of genotype and footshock was used to analyze c-fos immunolabeling data, the significance came out from genotype differences included all the testing animals (n = 16 vs n = 16).

**Figure 7:**
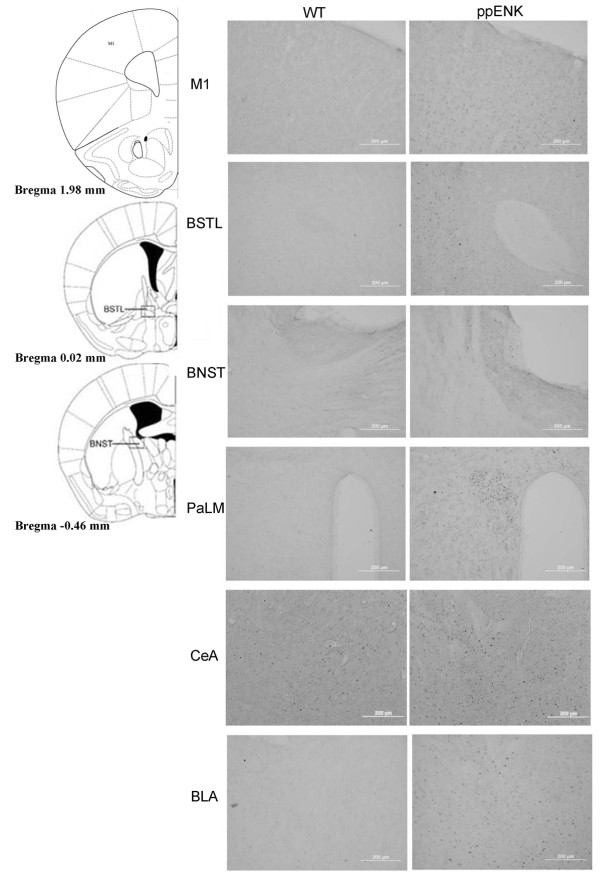
**Representative photomicrographs of significant Fos-LI after footshock in WT (left) and ppENK (right) mice**. M1, primary motor cortex; BSTL, bed nucleus of the stria terminalis-lateral division; BNST, bed nucleus of the stria terminalis-supracapsular division; PaLM, paraventricular hypothalamic nucleus-lateral magnocellular part; CeA central nucleus of the amygdala; BLA, basolateral nucleus of the amygdala.

Fos-like immunoreactivity (Fos-LI) was greater in ppENK mice compared with WT mice in the following brain areas: primary motor cortex (M1), bed nucleus of the stria terminalis-lateral division (BSTL), bed nucleus of the stria terminalis-supracapsular division (BNST), central nucleus of the amygdala (CeA), and basolateral nucleus of the amygdala (BLA) (*p *< 0.05) (Fig. 7, Table 1). Additionally, activation of the paraventricular hypothalamic nucleus-lateral magnocellular part (PaLM) was significantly greater in ppENK than WT mice (*F*_1,28 _= 3.54, *p *= 0.07). However, the following brain areas did not reach a significance difference between WT and ppENK mice: nucleus of ventral orbital cortex (VO), prelimbic and infralimbic cortex (PrL & IL), nucleus accumbens (AC), paraventricular thalamic nucleus (PVT), lateral hypothalamus (LH), cingulate/retrosplenial cortex (Cg/RS), medial nucleus of the amygdala (MeA), dentate gyrus (DG), CA1 field of the hippocampus (CA1), and CA2 field of the hippocampus (CA2) (*p *> 0.05) (Table 1).

## Discussion

The present results showed that different genotypes might appear different freezing responses. Footshock treatments had a significant effect. Also, there was a significant interaction between genotype and footshock. The present data mean the ppENK mice exhibited greater freezing responses compared with WT mice underlying a footshock treatment. The ppENK mice may be more sensitive to the aversive stimuli after receiving footshock treatments.

The four behavioral tests appeared to have genetic differences (i.e., genotype) and footshock effects. In the elevated plus maze test, ppENK mice took longer to reach the halfway point than WT mice. Footshock treatment induced a longer time to reach the halfway point and fewer entries into the open arms. However, in the light/dark test, ppENK mice did not show a significantly different latency to enter the dark chamber compared to WT mice. In the open field test, ppENK mice made fewer entries into the inner area compared with WT mice, and footshock was associated with fewer entries into the inner area. Moreover, ppENK mice spent less time in the inner area compared with WT mice. In the forced swim test, WT and ppENK mice did not exhibit significant differences in floating time or swimming time. In contrast, ppENK mice exhibited significantly less struggling time than WT mice. Footshock treatments had a significant effect on floating time and swimming time, but no significant effect was found on struggling time. Thus, these three depressive indices seemingly had inconsistent results in forced swim test. Taken together, the present study discovered that the enkephalin-deficient mice had augmented anxiety-like and depressive-like responses in multiple behavioral tests. The present data support the hypothesis that the endogenous enkephalins deficit might be prone to elicit anxiety- and depressive like PTSD symptoms.

The possibility that the brain opioid system mediates PTSD-like symptoms, such as traumatic stress or painful effects, is consistent with our findings showing that enkephalin-deficit mice have increased anxiety-like and depressive-like responses [16,18,19,36-38]. For example, when μ-opioid agonists are microinjected into the periaqueductal grey, locus coeruleus, nucleus raphe magnus, and nucleus reticularis gigantocellularis, an analgesic effect is observed [36]. Likewise, a prior study demonstrated that δ-opioid receptor agonists can elicit a spinal antinociceptive effect [19]. Moreover, functional neuroimaging data show that central μ-opioid receptors are activated when PTSD patients reexperience combat-related stimuli. In this previous study, cerebral blood flow was lower in the amygdala, AC, and dorsal frontal and insular cortex but higher in the orbitofrontal cortex [37]. When the effect of [D-Pen^2^, D-Pen^5^] enkephalin in spinal and supraspinal analgesia was tested, μ- and δ-opioid receptors were shown to mediate thermal analgesic, tail-withdrawal, and heat-induced tail-flick responses [18]. Thus, both μ- and δ-opioid receptors may have a crucial role in stress or pain.

Our behavioral data suggest that ppENK mice were more prone to anxiety- and depressive-like PTSD symptoms compared with WT mice, but few prior studies support this possibility [39]. For instance, pain responses, anxiety-like behavior, and aggressiveness have been shown to increase in ppENK mice [16]. Another study reported that the CeA inhibited the periaqueductal grey via the enkephalin system and suppressed affective defense responses [38]. A critical study demonstrated a negative correlation between β-endorphin-immunoreactivity in the central nervous system and PTSD-like symptoms [39].

Our Fos-LI data showed significant increases in the following brain areas in ppENK mice compared with WT mice: M1, BSTL, BNST, PaLM, CeA, and BLA. However, no significant differences were observed between WT and ppENK mice in the VO, PrL & IL, AC, PVT, LH, Cg/RS, MeA, DG, CA1, and CA2 (Table 1). We suggest that the M1, BSTL, BNST, PaLM, CeA, and BLA may be involved in enkephalins-regulated PTSD symptoms. The present Fos-IL data are partially consistent with previous studies though the neural substrates mediating PTSD-like symptoms remain uncertainty [40,41]. For example, the cellular data were demonstrated that after rats encountered the PTSD-like single-prolonged stress and intra-injected Lucifer Yellow into the BLA or CeA to analyze morphological changes, these authors found that the pyramidal neurons of BLA (but not CeA) were significant increase of dendritic arborization [42]. A recent review paper collects evidence on intrusive memories and dysfunction in declarative memory for human in past few decades, and proposes that the hippocampus, amygdala, and the prefrontal cortex are probably involved in the stress response of PTSD for human [40]. Neuroimaging research indicates that the medial prefrontal cortex, amygdala, sublenticular extended amygdala, and hippocampus maybe play a critical role in the PTSD-like dysregulation of emotional process [41]. Additionally, an adrenal gland lesion evidence have been manifested to not only decrease corticotrophin-releasing hormone-like immunoreactivity in BNST and CeA but also reduce corticotrophin-releasing hormone mRNA in the dorsal part of BNST, and thus the BNST and CeA are the part of extra-hypothalamus-pituitary gland-adrenal gland stress system that probably governs the PTSD-like fear and anxiety responses [43]. In conclusion, these brain areas are a part of the fear circuit and may be relevant to PTSD [37,42,43].

However, with regard to the relationship between subareas of the amygdala and PTSD-like symptoms, previous studies have yielded inconsistent results [38,42,43]. Cui et al. (2008) examined alterations in neuronal morphology and neurotransmitters in the CeA and BLA after rats were exposed to single, prolonged stress and found a significant enhancement of dendritic arborization in the BLA but not CeA. Thus, the authors suggested that only the BLA is involved in the traumatic stress of PTSD. By contrast, a recent study showed that adrenalectomy decreased corticotropin-releasing factor-LI in the CeA and BNST [43], suggesting that the BNST and CeA are a part of an adrenal steroid-sensitive extrahypothalamic circuit and regulate the fear and anxiety of PTSD-like symptoms. Furthermore, the CeA has been suggested to be a key structure involved in the inhibition of periaqueductal grey function via an enkephalinergic mechanism to control affective defense behavior [38]. Thus, the CeA and BLA have apparently different roles in PTSD. Nevertheless, the amygdala and BNST both are viewed to mediate anxiety- and fear-like PTSD symptoms.

Previously, some studies have found that opioids could attenuate the impact of the traumatic stress in affective and emotional states; suggesting that endogenous opioids (including enkephalin) maybe have a crucial role to regulate the stress responses including endocrine, autonomic nervous system, and fear behavior [15]. However, these studies of the endogenous opioid system did not provide a concrete hypothesis based on the effects of opioid function on PTSD-like symptoms. These studies remain in empirical investigations [7,12,13,44]. For example, when the placebo or an opioid antagonist naloxone is conditioned with a CS, the CS (regardless of pairing with placebo or naloxone) is demonstrated to increase pain tolerance [12]. Also, a report of human data indicate Vietnam veterans, who have PTSD-like symptoms in placebo condition but not naloxone condition, have significant decreases to pain perception after exposing combat videotape [13]. These previous studies suggest a centrally opioid response mediate PTSD-like stress or chronic pain. Based on our present data, we find that the ppENK mice are shown stronger anxious and depressive responses compared to WT mice. That may be due to ppENK mice have less pain threshold and/or more processing of pain perception. This issue needs to be scrutinized in the further study.

Nevertheless, we attempt to offer the oversensitivity hypothesis of enkephalin deficit-induced PTSD and suggest that the endogenous enkephalins deficit might be more sensitive to aversive stimuli such as the reexperiencing of traumatic events and avoidance of traumatic stimuli known to occur in PTSD. To illustrate, our data showed that the ppENK mice were more sensitive to anxiety-like and depressive-like responses, and these aversive two behaviors of ppENK mice were showing stronger than those of WT mice. Likewise, these two aversive behaviors were similar to those experienced by PTSD patients who exhibit associative fear conditioning. Interestingly, during the situational reminder sessions, the conditioned freezing response of the ppENK mice was stronger than those of the WT mice. Also, there was an interaction between genotype and footshock in the contextually conditioned fear behavior, besides four anxiety and depressive models. Thus, a major role of enkephalins might be to desensitize the magnitude of associative fear conditioning in PTSD-like symptoms, especially when patients reexperience traumatic stimuli. Conversely, a deficit in brain enkephalin levels may support the oversensitive responses observed in PTSD patients, such as anxiety and depressive responses.

In summary, the oversensitivity hypothesis of enkephalin deficit-induced PTSD provides a launching point for investigating the pathological mechanisms of PTSD. Multiple brain areas, such as the M1, BSTL, BNST, PaLM, CeA, and BLA, appear to be involved in the activation of endogenous enkephalins during the reexperiencing of traumatic events and avoidance of traumatic stimuli that characterize PTSD.

## Abbreviations

AC: nucleus accumbens; ANOVA: analysis of variance; BLA: basolateral nucleus of the amygdala; BNST: bed nucleus of the stria terminalis-supracapsular division; BSTL: bed nucleus of the stria terminalis-lateral division; CA1: CA1 field of the hippocampus; CA2: CA2 field of the hippocampus; CeA: central nucleus of amygdala; Cg/RS: cingulate/retrosplenial cortex; CS: conditioned stimulus; DG: dentate gyrus; Fos-LI: Fos-like immunoreactivity; IL: infralimbic cortex; LH: lateral hypothalamus; M1: primary motor cortex; MeA: medial nucleus of the amygdala; NGST: 3% normal goat serum containing 0.1% triton; PaLM: paraventricular hypothalamic nucleus-lateral magnocellular part; PBS: phosphate-buffered saline; PrL: prelimbic cortex; ppENK: preproenkephalin; PTSD: posttraumatic stress disorder; PVT: paraventricular thalamic nucleus; US: unconditioned stimulus; VO: nucleus of ventral orbital cortex; WT: wildtype.

## Competing interests

The authors declare that they have no competing interests.

## Authors' contributions

JCK, TCC, BCS, SH, and ACWH contributed to the design and conduct of the study, conducted the statistical analyses, drafted the manuscript and critically revised manuscript. All authors read and approved the final manuscript.
